# Application of ADA1 as a new marker enzyme in sandwich ELISA to study the effect of adenosine on activated monocytes

**DOI:** 10.1038/srep31370

**Published:** 2016-08-11

**Authors:** Chengqian Liu, Maksym Skaldin, Chengxiang Wu, Yuanan Lu, Andrey V. Zavialov

**Affiliations:** 1 Turku Centre for Biotechnology, University of Turku, Tykistokatu 6, 20520 Turku, Finland; 2 Tulane National Primate Research Center, 18703 Three River Road, Covington, LA 70433, USA; 3 Department of Public Health, John A. Burns School of Medicine, University of Hawaii at Manoa, 1960 East West Road, Biomed, Honolulu, HI 96822, USA

## Abstract

Enzyme-linked immunosorbent assay (ELISA) is a valuable technique to detect antigens in biological fluids. Horse radish peroxidase (HRP) is one of the most common enzymes used for signal amplification in ELISA. Despite new advances in technology, such as a large-scale production of recombinant enzymes and availability of new detection systems, limited research is devoted to finding alternative enzymes and their substrates to amplify the ELISA signals. Here, HRP-avidin was substituted with the human adenosine deaminase (hADA1)-streptavidin complex and adenosine as a detection system in commercial ELISA kits. The hADA1 ELISA was successfully used to demonstrate that adenosine, bound to A_1_ and A_3_ adenosine receptors, increases cytokine secretion by LPS activated monocytes. We show that hADA1-based ELISA has the same sensitivity, and also provides identical results, as HRP ELISA. In addition, the sensitivity of hADA1-based ELISA could be easily adjusted by changing the adenosine concentration and the incubation time. Therefore, hADA1 could be used as a detection enzyme with any commercial ELISA kit with a wide range of concentration of antigens.

Enzyme-linked immunosorbent assay (ELISA) is a very popular test that uses antibodies and the enzyme-mediated reaction to detect the antigen of interest in a fluid sample^1^,^2^,^3^. Among various types of ELISAs, a sandwich ELISA is highly specific, recognizing two different epitopes on the same antigen with two types of antibodies, i.e. a capture antibody attached to a solid phase, such as 96-well microtiter plates, and a detection antibody that is biotinylated^4^. In order to detect constituents present in low amounts, it is often necessary to devise procedures capable of strongly amplifying the enzymatic signal. Horseradish peroxidase (HRP) and alkaline phosphatase (AP), which were introduced in the 1970s, are the most commonly used enzymes to bind the detection antibody for a signal amplification^5^,^6^. Multiple chromogenic and fluorescent substrates were designed to detect the activity of HRP and AP in ELISA kits^4^. However, many of them are quite expensive, unstable, and potentially harmful. The last few decades have brought in new technology, both in large scale expression of recombinant enzymes and development of new detection systems, such as the UV spectrum plate readers. Adenosine deaminase (ADA, EC 3.5.4.4) is a key enzyme of the purine metabolism, catalyzing the irreversible deamination of adenosine or deoxyadenosine to inosine or deoxyinosine, respectively. ADA has been found in bacteria, plants, and animals^7^. In humans, there are two ADAs, hADA1 and hADA2^8^. Recently, we have developed a simple assay to detect hADA2 concentrations in biological fluids^9^. According to this assay, hADA2 is captured with polyclonal antibodies, which are absorbed by a 96–well microtiter plate, and then adenosine is added to detect ADA2 enzymatic activity. The amount of inosine produced is further analyzed on a separate detection plate, and can be followed by an increase in the absorbance ratio at 245 and 265 nm. The ratio is proportional to the hADA2 concentration, and hence, the concentration of ADA2 in a sample can be determined using hADA2 standards. In contrast to 120 kDA hADA2, hADA1 is a small 40 kDA enzyme, with higher turnover rate (*k*_*cat*_) and lower Km for adenosine^8^,^10^. Therefore, hADA1 and adenosine could be potentially used in the ELISA amplification system, applying a similar detection assay designed for hADA2.

To validate ADA1 ELISA, we studied the effect of low concentrations of adenosine on the cytokine secretion by monocytes in response to their activation with Lipopolysaccharides (LPS). It is known that the activation of human monocytes with LPS induces expression of A_1_, A_2A_, A_2B_, and A_3_ adenosine receptors^11^. According to the literature, adenosine is thought to be largely an immunosuppressive molecule^12^. It was shown that adenosine at a high concentration binds to A_2A_ receptors expressed on activated monocytes, and decreases the level of cytokines secretion, such as TNF-α^13^. However, little is known about the role of A_1_ and A_3_ receptors in the regulation of monocytes activity, and the effect of low adenosine concentration on the cells’ activation. Here, using selective and non-selective adenosine receptor antagonists, we investigated the effect of low-level adenosine in stimulation of cytokine secretion by LPS-activated monocytes. To measure the cytokine secretion, we used a standard ELISA with HRP or hADA1 as detection enzymes, and showed that hADA1 could substitute HRP in commercial ELISA kits.

## Results

### ADA1 expression and purification

**Figure 1:**
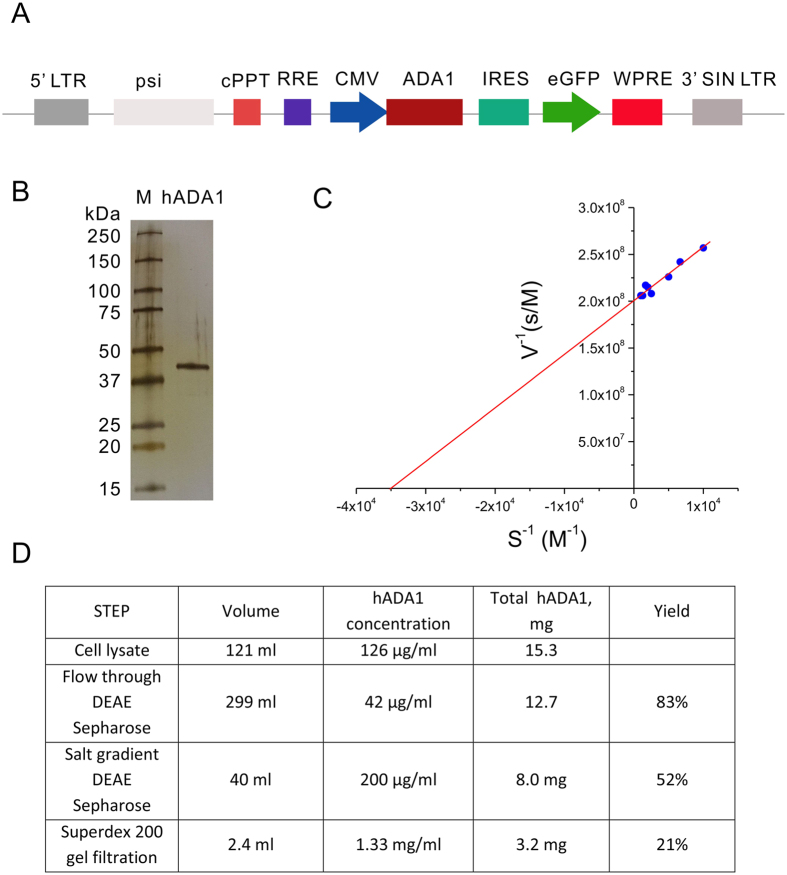
Expression and analysis of the human recombinant ADA1. (**A**) Lentiviral vector construct for hADA1 expression in mammalian cells; (**B**) SDS-PAGE analysis of purified hADA1: silver staining. Lane M-molecular weight markers, ADA1-purified human ADA1. Full-length gel is presented in Supplementary Figure 1. (**C**) Enzymatic analysis of the human ADA1 using Lineweaver–Burk plot. (**D**) Human ADA1 purification from 1.3 × 10^9^ 293T HEK cells.

Wild-type hADA1 was expressed in the cytoplasm of HEK 293T cells stably transfected with lentiviral particles, as described in Methods (Fig. 1A). The enzyme was purified with a high yield from the cell lysate using a simple two-step chromatography (Fig. 1D). SDS-PAGE analysis confirmed the high purity of the enzyme (Fig. 1B). Enzymatic analysis showed that hADA1 had similar catalytic parameters (k_cat_ = 190 s^−1^ and Km = 26 μM) to the recombinant hADA1 expressed in bacteria (Fig. 1C)^10^. Recombinant hADA1 was further chemically biotinylated without any loss of its enzymatic activity, indicating that the biotinylated hADA1 might be used to amplify the signals in standard ELISA.

### Design and sensitivity of ADA1 ELISA

**Figure 2:**
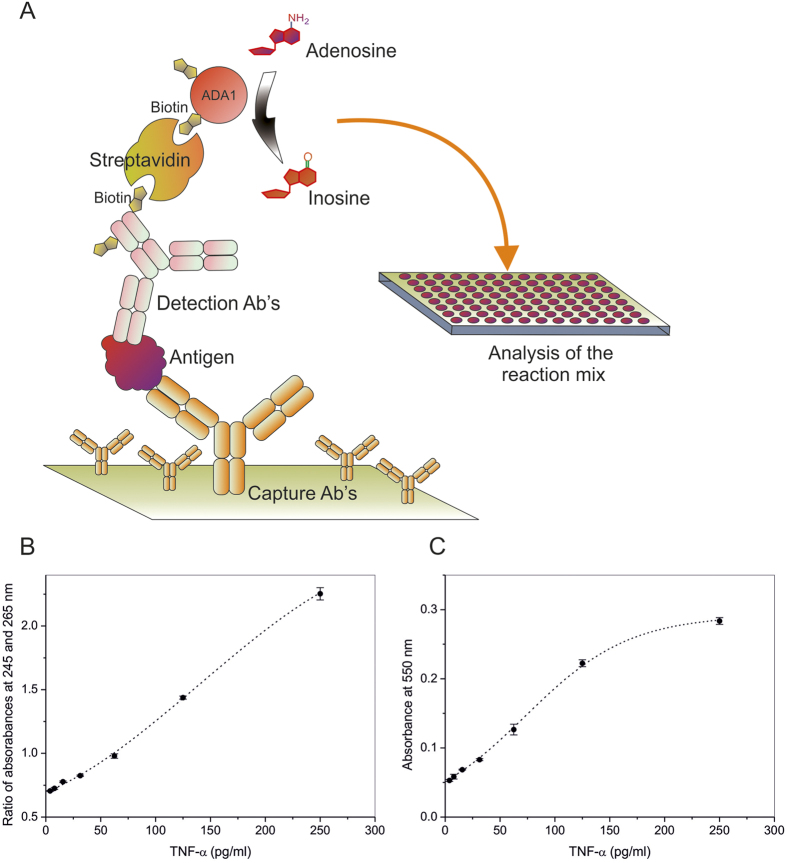
Design of hADA1 ELISA. (**A**) A schematic representation of the hADA1-based ELISA; (**B**,**C**) Standard curves for TNF- α, obtained using hADA1-streptavidin in standard ELISA. The concentration of TNF-α is proportional to the concentration of adenosine converted to inosine. (**B**) The reaction of adenosine hydrolysis to inosine by hADA1 is monitored by the absorbance ratio between 245 and 265 nm. (**C**) The amount of inosine produced is detected in the enzymatic color reaction, resulting in the increase in the absorbance at 550 nm. Each dot represents the average of two replicates.

**Figure 3:**
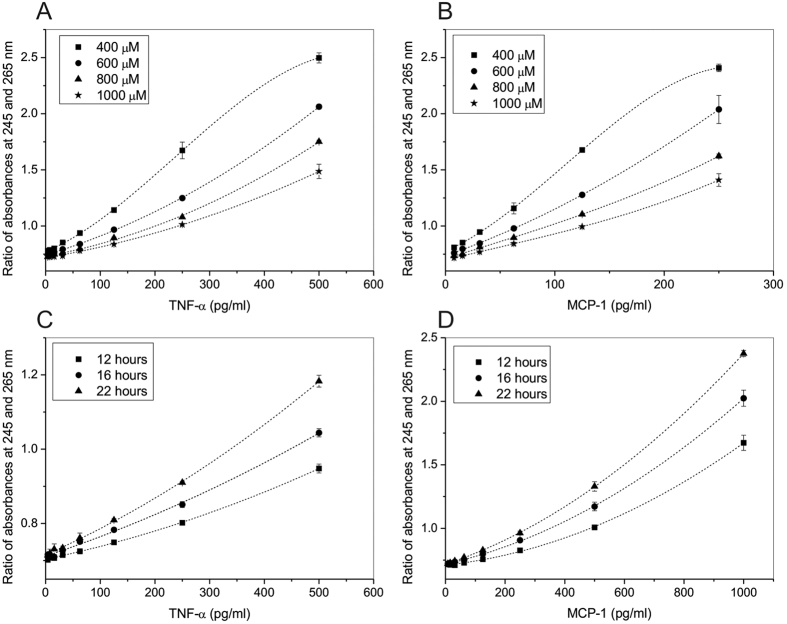
Sensitivity of hADA1 ELISA can be controlled by adenosine concentration and incubation time. (**A**,**B**) Standard curves obtained for TNF-α and MCP-1 ELISA at different concentrations of adenosine after 20 hours of incubation. (**C**,**D**) Standard curves obtained for TNF-α and MCP-1 ELISA at different times of incubation with 800 μM of adenosine after 20 hours of incubation. Each dot represents the average of two replicates.

**Figure 4:**
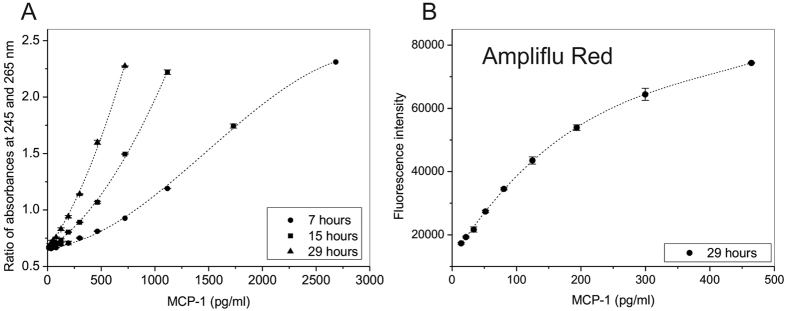
Human ADA1 ELISA can be applied to a wide range of antigen concentrations. (**A**,**B**) Standard curves obtained for MCP-1 ELISA after 29 hours incubation with 1 mM adenosine, and the following detection with Ampliflu Red fluorescent reagent. Each dot represents the average of two replicates.

**Figure 5:**
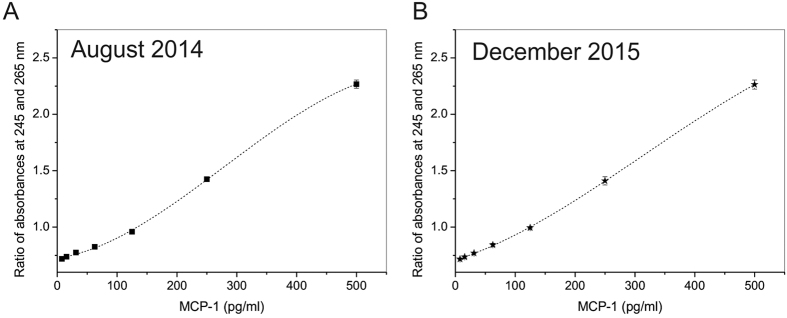
Stability of hADA1 and reproducibility of the results. Standard curves obtained for MCP-1 ELISA at different dates (**A**,**B**) with the same stock of biotinylated hADA1. Each dot represents the average of two replicates.

In the next series of experiments, we set up hADA1 ELISA using commercially available kits from BioLegend, to prove that hADA1 can be used as a detection enzyme. The antigen, bound to monoclonal capture antibodies, which were attached to the surface of 96-well microtiter plates, was further linked to biotinylated detection antibodies following a standard protocol of the ELISA kit (Fig. 2A). In the next step, hADA1-streptavidin complex was added to the plate. Subsequently, after the incubation with hADA1-Streptavidin, adenosine was added to start the reaction of adenosine deamination by hADA1. The reaction was stopped by a transfer of an aliquot of the reaction mix to a separate detection plate, and the concentration of inosine was detected using three different assays. The first, so-called UV assay, was originally developed to measure the concentration of hADA2 in human plasma^9^. Accordingly, the reaction mix from the first plate was transferred to a UV transparent 96-well plate, containing water to dilute the nucleosides to 0.1 mM concentration, and then the UV-transparent plate was read at 245 and 265 nm on a Synergy H1 hybrid Reader (Biotek). The reaction of deamination was followed by an increase in the ratio between the absorbances at 245 and 265 nm. The ratio was proportional to the concentration of hADA1 and antigen (Fig. 2B). This simple assay doesn’t require any additional reagents, and the reaction mix from the first plate can be analyzed multiple times (Fig. 3). Alternatively, in a color assay, the reaction of adenosine deamination could be monitored by a method wherein inosine is subsequently converted by purine nucleoside phosphorylase (PNP) and xanthine oxidase (XOD) to uric acid and hydrogen peroxide, which is further reacted with N-Ethyl-N-(2-hydroxy-3-sulfopropyl)-3-methylaniline (EHSPT) and 4-aminoantipyrine (4-AA) in the presence of peroxidase (POD) to generate quinone dye^14^. Quinone dye was detected at 550 nm in the visible spectrum, which allows using standard 96-well plates and a simple plate reader (Fig. 2C). In the third assay, the sensitivity of inosine detection could be significantly increased by substituting EHSPT and 4-AA with a fluorescent reagent, Ampliflu Red. Accordingly, Ampliflu red is converted into a highly fluorescent compound, resorufin, and the reaction of adenosine deamination is monitored by the increase in fluorescent intensity (Fig. 4B). The sensitivity of the hADA1 assay for a given ELISA kit or antigen concentration can be adjusted either by changing adenosine concentration or incubation times (Fig. 3). In general, the increase in the incubation time and the use of lower adenosine concentrations allow reaching the maximum sensitivity of the assay. Therefore, hADA1 ELISA could be used for a wide range of antigen concentration, ranging from nanograms to picograms per milliliter (Fig. 4A). The use of fluorescent reagents adds even higher sensitivity to the assay (Fig. 4B). Biotinylated human ADA1 is very stable and it could be kept at +4 ^o^C in a phosphate-saline buffer containing sodium azide. The enzyme remains fully active for an extended period of time providing reliable and reproducible results (Fig. 5).

### Study of monocytes activation in the presence of ADORs antagonists using a sandwich ELISA with either HRP or hADA1

**Figure 6:**
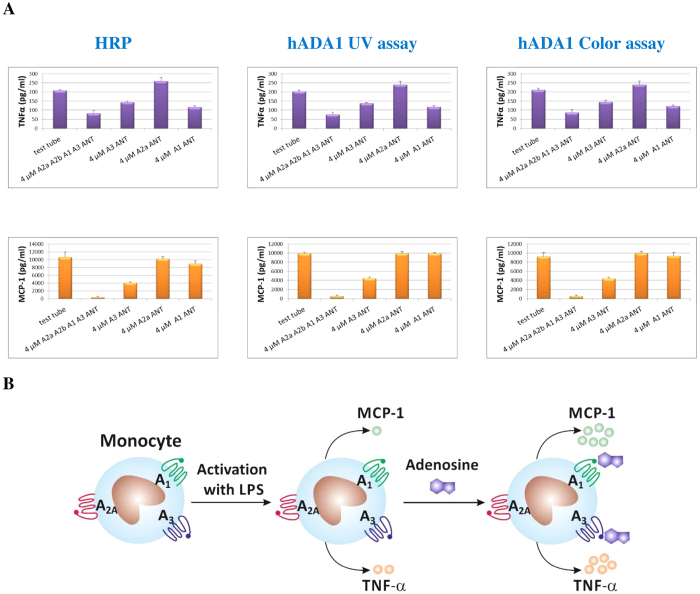
Effect of ADORs antagonists on TNF-α and MCP-1 secretion by LPS-activated monocytes. (**A**) Graphs showing the concentration of TNF-α and MCP-1 released by LPS-activated monocytes, which was determined either with a standard ELISA kit (HRP) or with hADA1 ELISA, using UV (Fig. 2B) and color (Fig. 2C) detection assays. The monocytes were incubated with 10 ng/ml LPS, either in the absence (test tube) or presence of nonselective A_2A_A_2B_A_1_A_3_ or selective A_3_, A_2A_ and A_1_ ADOR antagonists. (**B**) A schematic representation of the experiment.

It has been shown that monocytes activation is controlled by adenosine^15^. Here, we used LPS-activated monocytes to study the effect of different ADOR antagonists on the expression level of TNFα or MCP-1. To compare the hADA1-based ELISA system with a standard HRP ELISA, the concentration of cytokines in the cell supernatants were determined in parallel, using either HRP or hADA1 as a detection enzyme. As shown in Fig. 6A, the results obtained with HRP and hADA1 were almost identical. The addition of a non-specific adenosine receptor antagonist to the LPS-activated monocytes leads to a decrease in both TNF-α and MCP-1 secretion by the cells. This indicates that, in the absence of ADOR antagonist, adenosine, which is present in a test tube, binds to adenosine receptors and stimulates secretion of the cytokines by activated cells. Moreover, the release of MCP-1 by the cells is completely abolished when adenosine receptors are inactivated by ADOR antagonists. It is known that, at low adenosine concentration, only A_1_ and A_3_ receptors can bind adenosine^16^. The binding to A_2A_ receptor requires higher concentrations of adenosine, because this receptor has a lower affinity for the ligand. Thus, we used specific A_1_, A_3_, and A_2A_ ADOR antagonists to reveal the receptors that are involved in the regulation of monocytes activation. As seen in Fig. 6, the addition of A_2A_ antagonist didn’t affect the level of both cytokines secretion, suggesting that this receptor is not involved in the cells’ activation at low levels of adenosine. Conversely, A_3_ antagonist almost completely inhibited MCP-1 secretion by LPS-activated monocytes. There was a negative effect on TNF-α secretion as well. The effect of the A_1_ antagonist on MCP-1 release from the activated monocytes was negligible, while the level of TNF-α secretion was significantly diminished. These results show that, at low concentration, adenosine binds to A_3_ and A_1_ receptors, and stimulates the cytokines secretion by activated monocytes.

## Discussion

To date, a few enzymes have been employed as markers in the enzyme immunoassays^4^,^6^,^17^. Horseradish peroxidase, alkaline phosphatase, and β-galactosidase were the main enzymes employed to amplify signals in the enzyme-linked detection assays. One of the reasons for the scarce choice of the enzymes is the high requirements placed on them, such as a high specific activity of the enzyme after the labeling, availability of the purified enzyme at low cost, high stability, availability of inexpensive and stable substrates, and their stable chromogenic and fluorogenic products^4^. Horseradish peroxidase is ideal in many respects for these applications, because it is smaller, more stable, and less expensive than other popular alternatives, such as alkaline phosphatase. It also has a high turnover rate that allows generation of strong signals in a relatively short time span. Therefore, the majority of commercial ELISA kits utilize HRP as a detection enzyme. There are a variety of HRP substrates available for chromogenic, chemifluorescent, and chemiluminescent imaging. However, a low stability, nonspecific color precipitation and toxicity could be the main issues for the selected substrates. Another inconvenience is that each company tends to produce their own HRP conjugates and detection substrates, which are not interchangeable between the kits of different companies. A short range of the antigen concentration that could be detected with a given ELISA kit often results in the loss of valuable samples, because the concentration of the antigen in the samples doesn’t fit a standard curve. Here, we describe an alternative detection system, with human ADA1 as a detection enzyme, which has several advantages over HRP ELISA. The use of two separate plates for the antigen binding and the analysis of enzymatic reaction gives more flexibility, allowing the use of hADA1 ELISA kits with UV, fluorescent, and visible spectrum plates. It also allows achieving the desired sensitivity of the kit to get an accurate estimate of the antigen concentration in the sample.

At first, we developed a simple technology to produce large quantities of recombinant human ADA1 (Fig. 1D). The enzyme was expressed in 293T HEK cells transfected with lentiviral particles containing hADA1 gene (Fig. 1A). To the best of our knowledge, this is the first report on purification of fully active human recombinant ADA1 from mammalian cells. Human ADA1 is not toxic to the cells and, thus, it is accumulated at high concentrations in the cell cytoplasm. An easy three-step purification protocol permits isolating highly active and pure hADA1 (Fig. 1B). The chemical biotinylation of the enzyme didn’t affect the enzyme activity, which was preserved for at least one year (Fig. 5). In contrast to HRP, which is inhibited by sodium azide, hADA1 could be stored at 4^ o^C in PBS containing NaN_3_. Biotinylated hADA1 was simply coupled to streptavidin by mixing of two proteins. Since hADA1 is a small 40 kDa enzyme, the streptavidin-hADA1 complex can be bound to biotinylated detection antibodies without any steric hindrance (Fig. 2). The enzymatic reaction was started by adding a natural ADA substrate adenosine in PBS buffer. The main advantages of adenosine are that it is cheap, soluble, stable, and non-toxic. It could either be stored in solution or prepared freshly by dissolving in PBS. The reaction of adenosine deamination, catalyzed by hADA1, was stopped by transferring an aliquot of the reaction mix to a detection plate (Fig. 2A). Due to the difference in the absorption spectrum between adenosine and inosine, the reaction of deamination could be followed by the increase in the ratio of absorbances at 245 and 265 nm (Fig. 2B). This strategy was already successfully employed previously to monitor the activity of ADA2^9^. Alternatively, adenosine deamination could be detected in a cascade of enzymatic reactions, using either chromogenic (Fig. 2C) or fluorescent substrates (Fig. 4B).

The advantage of the hADA1 based ELISA is that the reaction could be analyzed on a separate detection plate at different times of incubation with adenosine (Fig. 3C,D). This adds more flexibility over the HRP system (Table 1). Therefore, it is a possible to adjust the sensitivity of the assay to get the highest accuracy for the antigen detection in the samples, by varying either adenosine concentration (Fig. 3A,B) or the incubation time with adenosine (Fig. 3C,D). This also allows adopting this system for use with any commercial ELISA kit. Moreover, the use of hADA1 instead of HRP permits the increase of the range of concentration of standards to detect the samples with a broad distribution of the antigen concentrations. For instance, the samples with high concentration of antigen could be read after a short incubation time, while the samples with low concentrations of antigens could be detected after a prolonged incubation with adenosine (Fig. 4A). Although it takes about 7 hours to reach a sensitivity comparable to HRP ELISA, the incubation time could be reduced to less than 1 hour by decreasing the concentration of adenosine 10 times. The detection diapason can be further extended by the use of Ampliflu Red fluorogenic reagent (Fig. 4B). Therefore, with the hADA1 detection system, there is no need to dilute samples to fit a narrow window of concentration, as required in the HRP-based ELISA kits. In addition, the completeness of the reaction of deamination can be checked by reading the standard curve together with a few key samples before the analysis of the whole plate. This procedure, which cannot be done with the HRP ELISA, increases the accuracy of the antigen detection and assures that the antigen concentration in all samples fits to the standard curve.

To validate hADA1 as a detection enzyme, we used both HRP and hADA1-based ELISA to measure the concentration of cytokines produces by LPS-activated monocytes in the cell culture medium. We studied the effect of low concentrations of adenosine, which accumulates in the cell culture, on the level of TNF-α and MCP-1 secretion by activated monocytes. To block the binding of adenosine to adenosine receptors (ADOR), we added either nonselective ADOR antagonist or selective A_1_, A_3_, or A_2A_ antagonists. In the presence of nonselective ADOR antagonist, we saw a dramatic decrease in the release of both cytokines by activated monocytes (Fig. 6A). Furthermore, the level of MCP-1 was almost undetectable. This indicated that adenosine was present in our system, and that the blocking of adenosine receptors with ADOR antagonists prevented adenosine binding to ADORs. We used specific ADORs antagonists to find out which of the ADOR receptors bind adenosine and stimulate cytokine secretion by activated monocytes. It was shown that blocking of A_2A_ receptor didn’t change the level of cytokines secretion into the culture medium, suggesting that the concentration of adenosine was too low to activate A_2A_ receptor. However, the blocking of either A_3_ or A_1_ receptor decreased the concentration of cytokines released by activated monocytes. In addition, our results showed that TNF-α secretion was stimulated by adenosine binding to both A_1_ and A_3_ receptors, while the level of MCP-1 secretion was mainly controlled by A_3_. This suggests that the binding of adenosine at low concentrations to either A_1_ or A_3_ receptors stimulates the release of TNF-α and MCP-1 (Fig. 6B). This effect is opposed to that of high concentrations of adenosine, where secretion of TNF-α by LPS-activated cells is inhibited by adenosine bound to A_2A_ receptors^13^ Therefore, a stimulatory effect of low concentrations of adenosine on the monocytes activation should be considered when ADOR agonists and antagonists are validated as potential drugs to tackle various immune disorders^18^. In our experiment, both hADA1 and HRP ELISA gave identical results (Fig. 5), proving that ADA1-based ELISA could be used as a valuable tool to measure antigen concentrations in biological fluids.

In conclusion, in this work we demonstrate that ADA1 can be used as an alternative detection enzyme in commercial ELISA kits (Table 1). The main advantage of the ADA1-based ELISA is the use of a stable, cheap, and non-toxic substrate adenosine. The product of the enzymatic reaction, inosine, has a different absorbance spectrum than adenosine, allowing the enzymatic reaction to be followed by a change in a ratio between absorbance at 245 and 265 nm^9^. The amount of adenosine produced can be detected on a separate detection plate at different incubation time points with adenosine using ultraviolet spectroscopy, colorimetry, and fluorescent assays. This adds flexibility to the assay, resulting in a higher accuracy and a broader range of antigen concentration detected by commercial ELISA kits.

## Methods

### Cloning and expression of the human ADA1

All methods were carried out in accordance with the relevant institutional guidelines. To stably express human ADA1 in cells transfected with a replication-defective lentiviral vector, the open reading frames (ORFs) of the genes were PCR amplified with the following primers: (F) 5′-AGCTCGAGACCGGTCCACCATGGCCCAGACGCCCGCCT-3′ and (R) 5′-ATGGATCCGCTAGCTCAGAGGTTCTGCCCTGCAG-3′ (hADA1). The PCR products were then subcloned into the pCR2.1-TOPO plasmid, excised by restriction digest with *Xho*I and *BamH*I, and ligated into a *Xho*I/*BamH*I-digested self-inactivating (SIN) transfer plasmid (pHR-cPPT-hB7-SIN)^19^. HEK 293T cells were then transfected with the transfer, VSV-G envelope and ΔR 8.2 packaging plasmids using the calcium phosphate transfection reagent^20^. Subsequently, lentiviral particles were purified by ultracentrifugation from the cell culture medium of these HEK-293T cells, and were used to infect new 293T cells. The recombinant human ADA1 was purified from the 293T cell lysates. The cells were grown in complete DMEM medium (Sigma-Aldrich), supplemented with 5% FBS, 100 U/ml penicillin, 100 μg/ml streptomycin, and 2 mM L-glutamine. The cells were trypsinized with trypsin/EDTA (Sigma-Aldrich), centrifuged for 5 min at 300 g, and the cell pellet was frozen in liquid nitrogen and kept at −80 ^o^C. Frozen 293T cells expressing hADA1 were lysed through three freeze/thaw cycles, using liquid nitrogen and a 42 ^o^C water bath. The cell lysate was re-suspended in 50 ml of ice-cold 50 mM Tris-HCl buffer (pH 6.8), 50 mM NaCl, 10 μM Zn(AcO)_2_, and 0.02% NaN_3_ (buffer A). The supernatant was separated from the cell debris by centrifugation at 4,000 g for 20 min, filtered using 0.45 μm filters (Sigma-Aldrich), and applied onto a DEAE Sepharose (GE Healthcare) equilibrated with buffer A. The flow through containing ADA1 was collected, and the pH was adjusted to 8.4 with Tris base. The enzyme was applied onto a DEAE Sepharose column equilibrated with buffer B (50 mM Tris-HCl pH 8.5, 10 μM Zn(AcO)_2_, and 0.02% NaN_3_). hADA1 bound to the column was eluted using 0–500 mM NaCl gradient. The fractions containing ADA activity were pooled, concentrated using 10 kDa centrifuge ultraconcentrators (Millipore), and further purified on a Superdex 200 column (GE Healthcare), equilibrated with phosphate-buffered saline (PBS) containing 10 μM Zn(AcO)_2_ and 0.02% NaN_3_. The kinetic parameters of the recombinant human ADA1 and its activity were determined as described before^8^,^9^. Purified hADA1 was chemically biotinylated using a kit from G-Biosciences.

### Monocytes isolation and culture

Human CD14 + monocytes were purified from buffy coats of healthy donors (Red Cross, Helsinki, Finland) by first isolating PBMCs with a Ficoll density gradient, and subsequently selecting for CD14 + monocytes using anti-CD14-conjugated magnetic microbeads (Miltenyi), as described previously (Zavialov *et al*., 2010a). The recovered cells were 95–99% CD14 + , as determined by flow cytometry using the FITC conjugated anti-human CD14 mAb (BD Biosciences). RPMI 1640, supplemented with 1% nonessential amino acids, 1% sodium pyruvate, 100 U/ml penicillin, 100 μg/ml streptomycin, 2 mM L-glutamine, 0.1% 2-mercaptoethanol, and 10% FBS, was used as the complete medium in the cell cultures of monocytes. Monocytes were cultured in suspension in 5 ml polypropylene tubes (Falcon) at 0.5 × 10^6^ cells/ml, in 0.5 ml of the culture medium. Monocytes were activated with 10 ng/mL LPS in the presence of either A_1_A_2A_A_2B_A_3_ antagonist CGS 15943, A_2A_ antagonist SCH 442416, A_1_ antagonist PSB 36, or A_3_ antagonist 2-phenyl-amino-N6-endo-norbornyladenine (Santa Cruz Biotechnology). After 20 hours culture in the tubes at 37 ^o^C, 5% CO_2_, the cells were separated by centrifugation (300 g, 5 min), and the supernatants were collected to measure TNF-α and MCP-1 concentration by ELISA (Bio Legends), with HRP or hADA1 as detection enzymes.

### ELISA using hADA1

The concentration of TNF-α or MCP-1 was determined using BioLegend ELISA kits according to the manufacturer’s instructions. For the experiments with ADA1-based ELISA, avidin-HRP was exchanged for hADA1-streptavidin complex following the protocol below:

1. Prepare 100 μg/ml hADA1-Streptavidin (based on the streptavidin concentration) by mixing hADA1 with Streptavidin in a molar ratio 0.5:1.
2. Dilute 100 μg/ml hADA1-Streptavidin to 500 ng/ml with 0.5% BSA, PBS, and 0.02% NaN_3_.
3. Add 100 μl of 500 ng/ml hADA1-Streptavidin to the wells of a 96-well plate, and incubate for 30 min at room temperature on a shaker.
4. Wash the plate 3 times with 200 μl 0.05% Tween 20 in PBS.
5. Wash the plate 1 time with 200 μl of PBS.
6. Add 100 μl of 800 μM adenosine in PBS, 5 μM Zn^2+^.
7. Incubate the plate at 37 ^o^C for 20 hours.
8. Transfer 25 μl of the reaction mix to a UV-transparent plate (Costar) containing 175 μl H_2_O, to make up 200 μl of 0.1 mM adenosine. Read the plate on a Synergy H1 hybrid Reader (Biotek) at 245 and 265 nm to get the 265/245 ratio, and determine the concentrations from a standard curve. The UV-transparent plate can be reused multiple times.

Alternatively, 40 μl of the reaction mix were transferred onto a 96-well cell culture plate (Greiner) containing 60 μl of 0.0625 U/ml purine nucleoside phosphorylase, 0.025 xanthine oxidase, 0.01 U/ml horse radish peroxidase, 1.25 mM 4-aminoantipyrine, and 0.75 mM N-Ethyl-N-(2-hydroxy-3-sulfopropyl)-3-methylaniline (all from Sigma-Aldrich) in 0.1 M Tris-HCl pH 6.8. The plate was incubated at 37 ^o^C for 30 min, and the absorbance at 550 nm was determined using a Synergy H1 hybrid Reader (Biotek). For the detection using a fluorescent reagent Ampliflu Red (Sigma-Aldrich), 1.25 mM 4-aminoantipyrine and N-Ethyl-N-(2-hydroxy-3-sulfopropyl)-3-methylaniline were substituted with 0.1 mM Ampliflu Red, and the fluorescent intensity (excitation 530 nm and emission at 590 nm) was determined using a Synergy H1 hybrid Reader (Biotek).

## Additional Information

**How to cite this article**: Liu, C. *et al*. Application of ADA1 as a new marker enzyme in sandwich ELISA to study the effect of adenosine on activated monocytes. *Sci. Rep.* **6**, 31370; doi: 10.1038/srep31370 (2016).

## Supplementary Material

Supplementary Information

## Figures and Tables

**Table 1:** The main differences between ADA and HRP ELISA.

	ADA	HRP
Standard range for a single experiment	10 pg/ml–5000 pg/ml*	7.8 pg/ml–500 pg/ml*
Coefficient of variation (CV%)	2.1–6.5*	4.8–11.7*
Composition and concentration of the substrate	0.5–1 μM adenosine	TMB and H_2_O_2_ (proprietary information)*
Light sensitivity of the substrate	no	yes
Long-term stability of the substrate	yes	no
Can be used with buffers containing sodium azide	yes	no
Reaction can be analyzed several times	yes	no
Reaction can be repeated	yes	no
Reaction can be analyzed with a UV reader	yes	no
A chromogenic or fluorescent substrate must be present in the reaction mix	no	yes
Interchangeability	Can be used with different commercial ELISA kit	Pre-optimized to work with the reagents in a particular kit

*The parameters are given for MCP-1 ELISA kit (BioLegends). Coefficient of variation was calculated from the values of 4 different samples with 6 replicates of the same assay.

## References

[b1] Engvall E. & Perlmann P. Enzyme-linked immunosorbent assay (ELISA). Quantitative assay of immunoglobulin G. Immunochemistry 8, 871–874 (1971). 10.1016/0019-2791(71)90454-x 5135623

[b2] Lequin R. M. Enzyme immunoassay (EIA)/enzyme-linked immunosorbent assay (ELISA). Clinical chemistry 51, 2415–2418, 10.1373/clinchem.2005.051532 (2005). 16179424

[b3] Schuurs A. H. & van Weemen B. K. Enzyme-immunoassay: a powerful analytical tool. Journal of immunoassay 1, 229–249, 10.1080/01971528008055786 (1980). 6785317

[b4] Porstmann T. & Kiessig S. T. Enzyme immunoassay techniques. An overview. Journal of immunological methods 150, 5–21 (1992). 10.1016/0022-1759(92)90061-w 1613258

[b5] Van Weemen B. K. & Schuurs A. H. Immunoassay using antigen-enzyme conjugates. FEBS letters 15, 232–236 (1971). 10.1016/0014-5793(71)80319-8 11945853

[b6] Avrameas S. Amplification systems in immunoenzymatic techniques. Journal of immunological methods 150, 23–32 (1992). 10.1016/0022-1759(92)90062-x 1613256

[b7] Maier S. A., Galellis J. R. & McDermid H. E. Phylogenetic analysis reveals a novel protein family closely related to adenosine deaminase. J Mol Evol 61, 776–794 (2005). 10.1007/s00239-005-0046-y 16245011

[b8] Zavialov A. V. & Engstrom A. Human ADA2 belongs to a new family of growth factors with adenosine deaminase activity. The Biochemical journal 391, 51–57 (2005). 10.1042/BJ20050683 PMC1237138 15926889

[b9] Zhou Q. . Early-onset stroke and vasculopathy associated with mutations in ADA2. The New England journal of medicine 370, 911–920, 10.1056/NEJMoa1307361 (2014). PMC4193683 24552284

[b10] Gracia E. . Human adenosine deaminase as an allosteric modulator of human A adenosine receptor: abolishment of negative cooperativity for [H](R)-pia binding to the caudate nucleus. Journal of neurochemistry, 10.1111/j.1471-4159.2008.05602.x (2008). 18680557

[b11] Chavez-Valdez R. . Caffeine modulates TNF-alpha production by cord blood monocytes: the role of adenosine receptors. Pediatric research 65, 203–208, 10.1203/PDR.0b013e31818d66b1 (2009). 19047957

[b12] Cekic C. & Linden J. Purinergic regulation of the immune system. Nature reviews. Immunology 16, 177–192, 10.1038/nri.2016.4 (2016). 26922909

[b13] Zhang J. G., Hepburn L., Cruz G., Borman R. A. & Clark K. L. The role of adenosine A2A and A2B receptors in the regulation of TNF-alpha production by human monocytes. Biochemical pharmacology 69, 883–889, 10.1016/j.bcp.2004.12.008 (2005). 15748700

[b14] Delacour H., Sauvanet C., Ceppa F. & Burnat P. Analytical performances of the Diazyme ADA assay on the Cobas(R) 6000 system. Clinical biochemistry 43, 1468–1471, 10.1016/j.clinbiochem.2010.09.005 (2010). 20850428

[b15] Hamano R. . Stimulation of adenosine A2A receptor inhibits LPS-induced expression of intercellular adhesion molecule 1 and production of TNF-alpha in human peripheral blood mononuclear cells. Shock 29, 154–159, 10.1097/shk.0b013e31812385da (2008). 17693933

[b16] Fredholm B. B. Adenosine, an endogenous distress signal, modulates tissue damage and repair. Cell Death Differ 14, 1315–1323, 10.1038/sj.cdd.4402132 (2007). 17396131

[b17] Porstmann B., Porstmann T., Nugel E. & Evers U. Which of the commonly used marker enzymes gives the best results in colorimetric and fluorimetric enzyme immunoassays: horseradish peroxidase, alkaline phosphatase or beta-galactosidase? Journal of immunological methods 79, 27–37 (1985). 10.1016/0022-1759(85)90388-6 3923120

[b18] Eltzschig H. K., Sitkovsky M. V. & Robson S. C. Purinergic signaling during inflammation. The New England journal of medicine 367, 2322–2333, 10.1056/NEJMra1205750 (2012). PMC3675791 23234515

[b19] Wu C. & Lu Y. High-titre retroviral vector system for efficient gene delivery into human and mouse cells of haematopoietic and lymphocytic lineages. J Gen Virol 91, 1909–1918, 10.1099/vir.0.020255-0 (2010). PMC3052536 20410313

[b20] Wu C. & Lu Y. Inclusion of high molecular weight dextran in calcium phosphate-mediated transfection significantly improves gene transfer efficiency. Cellular and molecular biology 53, 67–74 (2007). PMC2830788 17531163

