# Supplementary material for: Application of ADA1 as a new marker enzyme in sandwich ELISA to study the effect of adenosine on activated monocytes
Source: Sci Rep. 2016 Aug 11;6:31370. doi: 10.1038/srep31370 (PMC4980770; doi:10.1038/srep31370)
Supplement: Supplementary Information [file srep31370-s1.pdf]

Supplemental information

Application of ADA1 as a new marker enzyme in sandwich ELISA to study the effect of adenosine on activated monocytes

Chengqian Liu, Maksym Skaldin, Chengxiang Wu, Yuanan Lu, and Andrey V. Zavialov

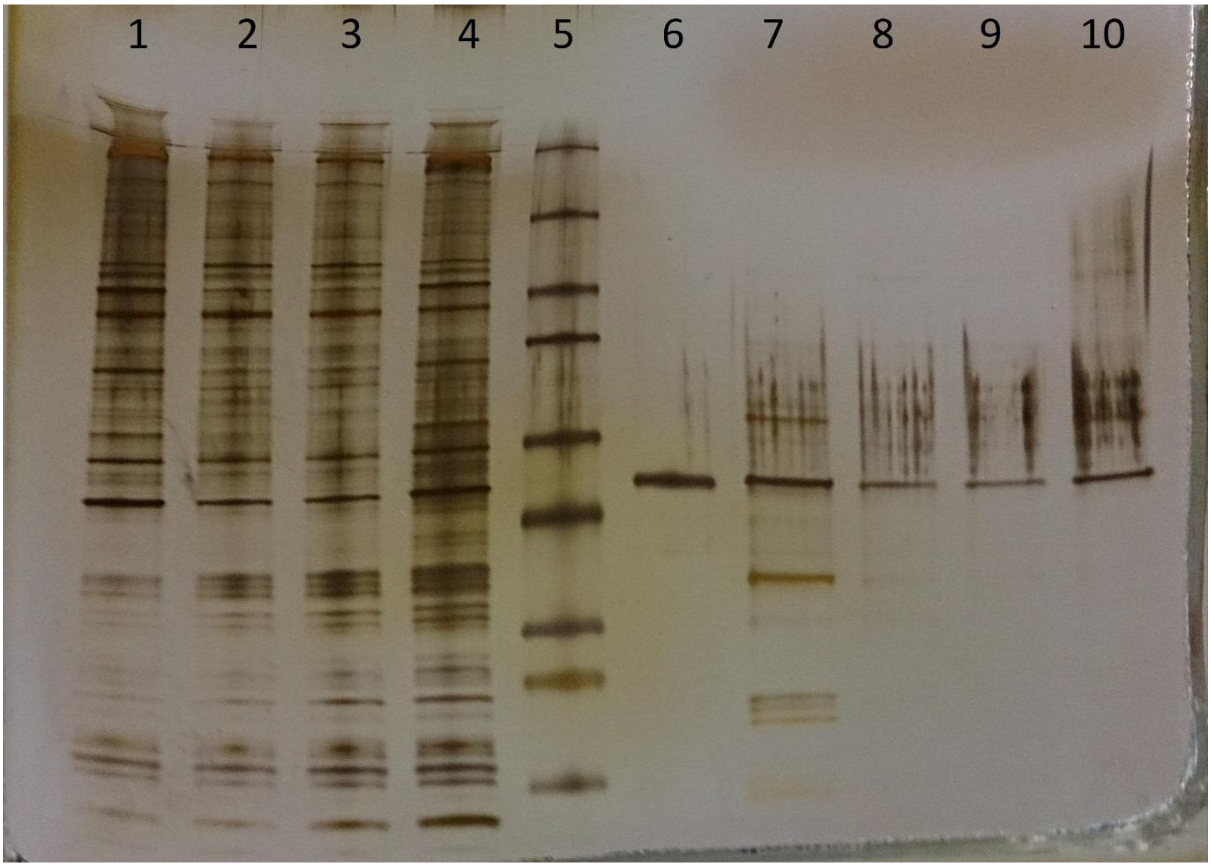

Supplementary Figure 1. SDS-PAGE analysis of purified hADA1: silver staining. Lane 5-molecular weight markers, Lane 6-purified human ADA1.
